# Without Mercy

**DOI:** 10.3201/eid2703.203690

**Published:** 2021-03

**Authors:** Jordi Casabona

**Affiliations:** Campus de Can Ruti, Badalona, Spain

**Keywords:** COVID-19, isolation, poetry, coronavirus disease, viruses

Between electric gloom,angels and demons dressed in plastic bluehave dragged me through the darkness,to the infinite black stone,where the origin and the end of time are lost.There, in the immense dark solitude,and in the imposed silence of word and thought,She, in my breath, gazed at mewith her eyes of fire,frosty and timeless,without any mercy,without any mercy.
